# Towards an Integrative Understanding of tRNA Aminoacylation–Diet–Host–Gut Microbiome Interactions in Neurodegeneration

**DOI:** 10.3390/nu10040410

**Published:** 2018-03-26

**Authors:** Elena L. Paley, George Perry

**Affiliations:** 1 Expert Biomed, Inc., 11933 SW 271st TER Homestead, Miami Dade, FL 33032-3305, USA; 2 Stop Alzheimers Corp., Miami Dade, FL 33032, USA; 3 Nova Southeastern University, 3301 College Ave, Fort Lauderdale, FL 33314, USA; 4 University of Texas at San Antonio, 1 UTSA Circle, San Antonio, TX 78249, USA; george.perry@utsa.edu

**Keywords:** tryptophan frequency, tryptamine in diet, protein biosynthesis, tryptophanyl-tRNA synthetase deficiency and gene mutations, neurodegeneration, tripeptides, Alzheimer’s disease, gut microbiome, vasculopathies

## Abstract

Transgenic mice used for Alzheimer’s disease (AD) preclinical experiments do not recapitulate the human disease. In our models, the dietary tryptophan metabolite tryptamine produced by human gut microbiome induces tryptophanyl-tRNA synthetase (TrpRS) deficiency with consequent neurodegeneration in cells and mice. Dietary supplements, antibiotics and certain drugs increase tryptamine content in vivo. TrpRS catalyzes tryptophan attachment to tRNA^trp^ at initial step of protein biosynthesis. Tryptamine that easily crosses the blood–brain barrier induces vasculopathies, neurodegeneration and cell death via TrpRS competitive inhibition. TrpRS inhibitor tryptophanol produced by gut microbiome also induces neurodegeneration. TrpRS inhibition by tryptamine and its metabolites preventing tryptophan incorporation into proteins lead to protein biosynthesis impairment. Tryptophan, a least amino acid in food and proteins that cannot be synthesized by humans competes with frequent amino acids for the transport from blood to brain. Tryptophan is a vulnerable amino acid, which can be easily lost to protein biosynthesis. Some proteins marking neurodegenerative pathology, such as tau lack tryptophan. TrpRS exists in cytoplasmic (WARS) and mitochondrial (WARS2) forms. Pathogenic gene variants of both forms cause TrpRS deficiency with consequent intellectual and motor disabilities in humans. The diminished tryptophan-dependent protein biosynthesis in AD patients is a proof of our model-based disease concept.

## 1. Introduction

Alzheimer’s disease (AD) clinical trials have been a tremendous investment loss. Efforts to combat neurodegeneration associated with AD are hindered by a lack of animal models recapitulating disease. The main reason is that the transgenic mice used for AD preclinical experiments clearly do not recapitulate the etiology of human disease. The failed clinical trials indicate that a cause of AD is still unknown. Animal models can certainly be useful to find out more about the biological bases of AD and develop efficient pharmacological treatments. The ethologically-based mouse models of AD would be extremely useful for new preclinical trials. The neuronal loss in brain is a main characteristic of AD. The neurofibrillary tangles (NFT) containing tau protein along with amyloid β (Aβ) plaques are the pathological features of AD brain. Although the relationship between the primary structure and function of the AD hallmarks tau protein and amyloid precursor protein (APP) has been relatively well studied, there are, to our knowledge, no structure-function analyses of biosynthesis for these proteins and other proteins implicated in neurodegeneration. Critics of the “amyloid hypothesis”, which posits that the AD is triggered by a build-up of Aβ in the brain, say the results of the failed clinical trials are evidence of its weakness [1]. Here we explore how Aβ and tau could become overrepresented in brain of AD and related diseases. In this report, we analyze results supporting the alternative hypothesis of “impaired protein biosynthesis causing neurodegeneration”. We used the basic local alignment search tool (BLAST) of the National Center for Biotechnology Information (NCBI) to find and summarize the evidence emphasizing the critical role of tryptophan content in proteins for the disease development. Here, we analyze the alterations in short peptide profiles [2] as an early indication of neurodegeneration in humans, which support our experimental cell and animal models of neurodegeneration induced by the natural inhibitors of protein biosynthesis [3,4,5]. These competitive inhibitors of a key enzyme of protein biosynthesis tryptophanyl-tRNA synthetase (TrpRS) are tryptophan metabolites tryptamine and tryptophanol [6,7] that can be produced by human and mammalian gut microbiota [8,9]. The tryptamine producing staphylococci were present in 60% of the human stool samples [10]. These bacteria possess the sadA gene, an aromatic amino acid decarboxylase that catalyzes conversion of tryptophan to tryptamine. The tryptamine production under aerobic conditions varied in different Staphylococcal strains from 71 ± 6 to 231 ± 10 mg/L. Tryptamine increases the adherence and internalization of staphylococci into human cells. The cytotoxic effect of tryptamine, but not of other trace amines tyramine and phenylethylamine, was demonstrated in human cells [10]. In colonocytes—the epithelial cells of colon—tryptamine is a selective modulator of the aryl hydrocarbon receptor (AhR), a ligand-activated transcription factor [11]. Investigation of the urine metabolic profiles showed significant increases in the concentrations of microbial tryptamine in probiotic supplemented mice [12]. Tryptamine produced also at the range 445 to 1157 mg/L by yeast strains isolated from the cow’s cheese in Spain [13]. The fungal pathogen *Fusarium* of bread wheat [14] and chickpea [15] produce tryptamine. The fungal microbiota (human gut mycobiome) was studied in 16 faecal samples from healthy humans with a vegetarian diet. Fungi were detected in each sample while *Fusarium* was the most abundant genus [16]. Tryptamine is increased under sporulating conditions in wheat pathogenic fungus *Stagonospora nodorum* [17]. The gut microbiome is altered in AD patients [18,19]. The gut microbiome of AD participants has decreased microbial diversity and is compositionally distinct from control age- and sex-matched individuals [19]. Our study revealed the gut bacterial sequence specific for AD patients [18]. This sequence belongs to the gene encoding Na(+)-transporting NADH: Ubiquinone reductase (NQR) while both the NQR substrate ubiquinone and Tryptophan are the products of Shikimate pathway. Both tryptamine and tryptophanol prevent the formation of adenylyl-enzyme [6], the intermediate (synonym adenylate-enzyme) in the reaction of tRNA aminoacylation [20]. In this report, we revisited our data to analyze them together with other related publications to shed light on different aspects of AD modelling. This approach prompts us to suggest the model-based disease concept that (1) the tryptophan metabolite tryptamine present in different dietary products in different geographical locations [21,22,23,24,25], produced by human gut microbes [10], inhibit the host protein biosynthesis via the TrpRS deficiency that leads to neurodegeneration and cell death in human organs [4]; (2) the microbial production of tryptamine can be modified by human diet and dietary supplements that can cause significant increase (six-fold) in excreted tryptamine [26]; (3) prescription drugs—antidepressants ( inhibitors of monoamine oxidase (MAO catabolizing tryptamine to indole-3-acetic acid (IAA)) potentiate the tryptamine activities causing seizures and death in animals [27]; (4) usage of antibiotics increases the tryptamine production in animals [9,28]; and (5) the levels of tryptamine (~10 mg/kg) is higher than those of tryptophan (~1 mg/kg) in transgenic potato tubers expressing tryptophan decarboxylase [29]. TrpRS activities can also be altered in vivo because (i) TrpRS is the interferon-inducible protein [30]; (ii) TrpRS is a secreted dietary protein that can be consumed with a cow milk [31,32,33]; (iii) TrpRS is a human autoantigen and anti-TrpRS autoantibodies bind both human and bovine TrpRS [34]; (iv) TrpRS expression is modulated by hypoxia [35]. This report also includes our experimental data on (1) TrpRS in AD vasculopathies revealed with the monoclonal antibodies (mAb) to TrpRS and (2) tryptamine-induced vasculopathies in the mouse model.

## 2. Materials and Methods

### 2.1. BLAST for Short Peptides Altered in Mild Cognitive Impairment and Alzheimer’s disease

We used BLAST for analysis of selected peptides to reveal the functions of proteins possessing tripeptides altered at the different stages of neurodegeneration, both mild and severe forms [2]. To this end, we designed the peptide sequences of 4 or 5 amino acids that included an altered tripeptide of interest as a search strategy in BLAST. The Tables S1–S4 summarize the results of the conducted search. The analysis of the altered Trp-containing peptides is included in the Tables S1 and S2, while the altered peptides lacking Trp are in the Tables S3 and S4.

### 2.2. Search for Tryptophan-Free Proteins

The Trp content in the selected proteins and polypeptides with a link or putative link to neurodegeneration was verified using Standard Protein BLAST of the NCBI databases and “Find” function of Microsoft Word to search for Trp (W) in the protein sequence of interest.

### 2.3. Histochemical Analysis of Vasculopathies

The treatment of mice with tryptamine at the concentrations that inhibit TrpRS, histochemical analysis and immunostaining of brain serial sections of AD, controls and mice were conducted as previously described; the monoclonal antibodies (mAb) and polyclonal antibodies (pcAb) to TrpRS are characterized and mapped [3,36].

## 3. Results and Discussion

### 3.1. TrpRS Inhibition and Aggregation Can Occur Physiologically

TrpRS is responsible for the presence of Trp in proteins. Here, we evaluate the TrpRS inhibition by natural compounds in vitro and in vivo. In the initial step of protein biosynthesis, TrpRS catalyzes the attachment of Trp to its cognate tRNA^trp^ with formation of tryptophanyl tRNA^trp^. We demonstrated the substrate inhibition (50–90%) of human TrpRS by Trp at physiological concentrations 10–50 µM [3]. Trp is an essential amino acid with *K*_m_ for purified bovine pancreatic TrpRS 1.4 ± 0.2 × 10^−7^ M in Trp-dependent ATP–PP_i_ exchange reaction [37]. The Michaelis constant *K*_m_ is the substrate, such as tryptophan concentration at which the reaction rate is half of V_max_, denoting a dose of a naturally occurring agent such as tryptophan that is within the range of concentrations or potencies that would occur naturally. The 10–50 µM physiological range refers to the naturally accurred Tryptophan, which includes its participation in a number of activities as a precursor, substrate and a product. Tryptophan as well as tryptamine occurs in the human body as the free and bound forms. A portion of the free tryptophan is involved in the de novo protein biosynthesis. The estimated tryptophan/tryptamine ratio does not reflect the exact in vivo status and depends on the methods of extraction and determination. The TrpRS substrate inhibition by Trp seems plausible because the Trp substrate inhibition was also demonstrated for other human enzymes implicated in tryptophan metabolism, such as Indoleamine 2,3-Dioxygenase [38,39] and Tryptophan Hydroxylase [40]. Moreover, Trp inhibits an enzyme specified by the tryptophan operon: anthranilate synthetase in bacteria [41] and plant [42]. Another substrate of the enzyme, tRNA can also inhibit mammalian TrpRS at the 2.9 µM Trp concentration [43]. The formation of fibrillary tangles of filaments resembling those in AD brain was detected in non-neuronal kidney cells [44], neuronal human cells and mouse brain [3] following treatment with TrpRS inhibitors (Table 1) tryptamine and tryptophanol [6,7] at the range of concentrations that can be available physiologically with the human dietary products [21] and excreted by healthy humans [8]. The levels of soluble and insoluble TrpRS forms and activity were altered following tryptamine and tryptophanol treatment in non-neuronal and neuronal cells [3,44,45].

The aggregated TrpRS was revealed in the tryptamine-treated cells and in Alzheimer’s postmortem brain [36]. TrpRS is prone to self-aggregation [47] and fibril formation [36] that cause enzyme inactivation. TrpRS self-aggregates in vitro under the enzyme concentrations higher than 10–15 µM or ~1 mg/mL [48]. TrpRS can be overexpressed in vivo intracellularly and extracellularly since this enzyme is the interferon-inducible protein [30] that is rapidly secreted upon pathogen infection [49].

### 3.2. Mechanism of TrpRS Inhibition by Tryptamine and Tryptophanol

Tryptamine is a competitive inhibitor of bovine pancreatic TrpRS [50]. Incubation of Trp (1.8 mM) and [β-^18^0_2_]ATP with purified mammalian TrpRS in the presence of Mg^2+^ for 21 h at 37 °C led, as expected, to complete positional isotope exchange. Incubation of [β-^18^0_2_]ATP and Mg^2+^ and TrpRS in the absence of added Trp for 21 h under the same experimental conditions also led to complete positional isotope exchange, and this could be attributed to endogenous Trp present in the enzyme preparation. Tryptamine and tryptophanol are potent competitive inhibitors of l-Trp for TrpRS, the *K*_i_ for tryptamine being 6.0 × 10^−7^ M (Appendix A). Incubation of the enzyme with 6 mM tryptamine , ATP and Mg^2+^ or 6 mM dl-tryptophanol, ATP and Mg^2+^ for 21 h at 37 °C gave no evidence of positional isotope exchange in the recovered ATP. These results show that an adenylyl-enzyme—the intermediate in the reaction of tRNA aminoacylation—is not formed between MgATP and TrpRS in the presence of tryptamine or tryptophanol [6]. Binding of Trp, tryptamine and ATP to TrpRS was studied by equilibrium dialysis experiments. There are two binding sites per mole of enzyme both for Trp and for tryptamine. The dissociation constant Ks for Trp is 0.95 μM and for tryptamine is 1.8 μM [48]. No cooperativity between the TrpRS subunits for the binding of the amino acid or of the competitive inhibitor is evidenced by equilibrium dialysis experiments. Therefore, TrpRS is roughly two-fold more tightly bound to Trp than to tryptamine. It was demonstrated that [14C]tryptophan bound at the tryptophanyl-adenylate active site of the bovine pancreatic TrpRS was completely removed from the enzyme by incubation with an excess of [^3^H]tryptamine [51].

### 3.3. Tryptamine and Tryptophanol Cytotoxicity and Bioavailability

Tryptamine induces cytotoxicity/neurotoxicity, neurofibrillary tangles, autophagic, axonal and mitochondrial pathologies and amyloidosis [3,5,52]. These characteristics are similar to those reveled in AD. Tryptophanol that also induced formation of fibrillary tangles in kidney cells is less toxic than tryptamine [44]. As a biogenic amine, tryptamine is enzymatically produced by decarboxylation of Trp. Tryptamine is catabolized to indole-3-acetic acid (IAA) by monoamine oxidase (MAO). Therefore, bioavailability of tryptamine depends on the levels and enzymatic activities of decarboxylase and MAO enzymes. The pathway for tryptophanol remains unknown. In our studies, mice had a diet with roughly two milligrams of total tryptophan per day (~0.2 mg of free tryptophan). Tryptamine that was injected intraperitoneally (each injection of 200 μg of tryptamine for 2.5 weeks was administered for every second day) induced neurodegeneration with the behavioral changes in mice [3].

### 3.4. Tryptamine and Tryptophanol Increases Following Antibiotic Treatment

Profiling study of urinary and fecal metabolites in Wistar rats exposed to a broad spectrum β-lactam antibiotic, Imipenem/cilastatin sodium demonstrated significant increase of tryptamine (up to four-fold in feces, 13-fold in urine) and tryptophanol (>51-fold in feces) along with Trp alterations in a time-dependent manner [9].

Early antibiotic intervention with in-feed antibiotics (olaquindox, oxytetracycline calcium and kitasamycin) from the postnatal day 7 to day 42 of pigs increased tryptamine concentration in the feces [28].

### 3.5. Dietary Exposure of Human Population to Tryptamine

In a recent report, the risk assessment of dietary exposure to tryptamine was analyzed for the Austrian population [24]. For fresh/cooked fish, preserved fish, cheese, raw sausage, condiments, sauerkraut and fermented tofu, maximum tolerable levels of tryptamine were 1650; 3200; 2840; 4800, 14120; 1740; and 2400 mg/kg, respectively. This assessment did not take into account the combined effects of simultaneously ingested biogenic amines, and increased susceptibility to tryptamine, e.g., due to reduced MAO activity, did not estimate prolonged effects of tryptamine consumption and did not consider the tryptamine content in individuals. Tryptamine was substantially higher (4.43-fold) in vaginal tract of smokers compared to non-smokers [53]. Smoking is a significant risk factor for AD [54].

### 3.6. Concentration-Dependent Tryptamine Effects in Animal Experiments: Seizures, Death

For comparison, we provide here the tryptamine concentrations used in the experiments with animals. In the mouse model of neurodegeneration, we observed convulsions following intravenous (i.v.) injections of one milligram tryptamine per mouse of ~25 g or ~40 mg/kg [3,4,5]. Tryptamine was utilized as an inducer of seizures in laboratory animals (i.v. administration of tryptamine at 20 mg/kg to rats) for the anti-seizure drug development by the pharmaceutical companies [55]. The potentiation of tryptamine toxicity on mice was studied with and without prior administration of MAO inhibitors, which are currently in use as antidepressants [27]. The LD_50_ for tryptamine hydrochloride injected subcutaneously in mice was found to be ~500 mg/kg, while death occurred without any marked symptoms of central stimulation. After subcutaneous administration of a MAO inhibitors nialamide or phenelzine, the LD_50_ of tryptamine was reduced from 500 to 85 mg/kg. Furthermore, the mice showed pronounced symptoms of central stimulation, including tremor, convulsions, and marked agitation [27]. Chronic administration of monoamine oxidase inhibitor antidepressant drugs (daily doses: tranylcypromine.HCl, 0.5 and 1.0 mg kg^−1^; phenelzine sulfate, 5 and 10 mg kg^−1^, each for 28 days; clorgyline.HCl, 1.0 mg kg^−1^; (−)-deprenyl.HCl, 1.0 mg kg^−1^, each for 14 days) resulted in decreases in [^3^H]tryptamine binding site density in brain cortical membranes from male Sprague–Dawley rats [56]. These results can indicate that unlabeled endogenous tryptamine is accumulated in brain following administration of the antidepressant drugs. Tryptamine easily crosses blood-brain barrier [57]. Tryptamine half-life depends on the activities of MAOs in the different body compartments. The tryptamine half-life in human or animal tissues of different organs including brain reflects tryptamine degradation by MAO-A, MAO-B [57] and diamine oxidase [58] during postmortem and extraction periods. In the author’s previous study of the radiolabeled tryptophan and tryptamine in the kidney cultured cells, the uptake for tryptamine exceeds the tryptophan uptake at 2.1–2.4-fold in both original and tryptamine resistant cells during at least two hours [4]. The half-life for TrpRS was determined to be less than two hours in human HeLa cells [59]. Despite the short half-life (1.6 min in the rat spinal cord [60]), the concentration of tryptamine can be high within a short period of time as a result of its high turnover [57]. Tryptamine is a precursor of the hallucinogen *N*,*N*-Dimethyltryptamine (DMT), an endogenous sigma-1 receptor regulator [61]. Administration of DMT in combination with MAO inhibitor can induce mania and psychosis [62]. A method of in vivo tryptamine monitoring in the brain and other organs does not currently exist.

### 3.7. Tryptamine in Healthy Human Population and in Diseases

The tryptamine production is determined to be varied in human gut of healthy subjects (*n* = 68) with overage tryptamine content in stool samples of 2.00 ± 1.24 µmol/g dry matter (~2.00 ± 1.24 mM) [8]. Quantitative abundance metabolomics profiles for human fecal metabolites include tryptamine with ~100-fold maximum difference among individuals from Oklahoma [63]. The free tryptophan content in normal human plasma of 5.5 μM (approx 1.3 mg/L) in males and 5.9 μM in females was similar in younger and older groups [64]. Metabolomics revealed the elevated urinary tryptamine excretion (3.3-fold) in Parkinson’s disease (PD) [65]. The author suggests here that the reason for the tryptamine elevation is the tryptamine-induced neurodegeneration in PD. High tryptamine levels (2.78 µg/g) detected in cataractous lenses [66] correlates with 1.43-fold increased risk of developing AD by older people with cataract [67] and 26% increased hazard of PD in cataract patients [68]. After an oral dose of normal human feces in the germ-free mice, the feces become the major route of the tryptamine elimination [69].

### 3.8. Tryptamine Upregulates Transcription of Genes Including Gene Encoding Aβ Precursor

The 22 transcripts were upregulated in mouse Hepa cells by tryptamine treatment at 50 μM for 6 h [70]. In this study, the 10 of 22 transcripts were up-regulated at 2 to 38.24-fold including the gene (4.10-fold) encoding Aβ precursor-like protein 2 (Aplp2), which has been implicated in AD pathogenesis [70]. The genes encoding Cytochrome P4501a2 (Cyp1a2) activated via aryl hydrocarbon receptor (AHR); and apoptotic protease activating factor (Apaf1) were upregulated by tryptamine at 38.24-fold and 2.23-fold, respectively. This study links our model of the tryptamine-induced neurodegeneration with Aβ hypothesis of AD.

### 3.9. The Diet Additives Increase Tryptamine Content in Animals

In the distal colon contents (dcc) of porcine neonates, the bacterial tryptamine was higher with formula diet (tryptamine at ~75 µg/mg of dcc) relative to sow diet (tryptamine at ~45 µg/mg of dcc) [71]. The diet fiber additives (4%) fructooligosaccharides (FOS, fructan) and pectin increased roughly six-fold the fecal tryptamine (1.17 µmol/g, cellulose control, 4% of diet) in adult cats [26]. In dogs, FOS (0.5% of diet) increased the fecal tryptamine (2.11 µmol/g versus 1.53 µmol/g in control) [72]. A 2.5-fold increase was observed in the tryptamine fecal concentrations following feeding fructan inulin (3 g/kg of body weight per day) to normal ponies [73]. In a rumen fluid of the ruminant animal goat, the metabolomics revealed that a high grain feeding (50% maize) increased the levels of tryptamine to 284.08 µM compared to control (145 µM tryptamine) with no maize feeding [74].

Note that the diet additives increasing tryptamine levels in animals inulin and oligofructose are the regular components in the diets of Americans [75] and pectin, which is an abundant polysaccharide in the human diet is metabolized by gut bacteria [76].

### 3.10. Other Trp and Tryptamine Metabolites Can Inhibit TrpRS

In this section, we discuss the possibility that other Trp metabolites, including products of tryptamine degradation can be implicated in neurodegeneration and other diseases via TrpRS inhibition. Table 1 displays the TrpRS inhibitory analysis of different Trp metabolites.

#### 3.10.1. IAA

The product of tryptamine degradation IAA (synonyms: beta-Indolylacetic acid, indolylacetic acid) is also a TrpRS inhibitor [46] although less potent than tryptamine. IAA is a uremic toxin derived from Trp fermentation by gut microbiota; it accumulates in patients with chronic kidney disease on haemodialysis [77]. IAA upregulates the eight genes regulated by the transcription factor aryl hydrocarbon receptor (AHR) [78]. The reduction in tryptophan metabolism, but the activation of IAA and AHR were revealed in the gut microbiota of individuals with inflammatory bowel disease [79]. Note, tryptamine forms the two-fold chiral helixes [80] with IAA in vitro. IAA is a plant hormone of the auxin class. IAA production is widespread among environmental bacteria that inhabit soils, waters, but also plant and animal hosts. IAA was detected in mammalian feces [9]. IAA was measured in human plasma of 15 healthy individuals at 0.61–3.32 µmol/L [81]. In the recent study, IAA and Trp were detected in feces of healthy subjects at ~2–12 nmol/g and ~100 nmol/g respectively [79]. Effect of eight antibiotics on the formation of IAA from Trp by ruminal microorganisms was studied: seven of eight antibiotics increased IAA [82]. We conclude that the product of tryptamine degradation by MAO can be also involved in protein inhibition in vivo.

#### 3.10.2. Indolepyruvic Acid

TrpRS inhibitor, indolepyruvic acid (Table 1) is the product of catalysis of Trp by Trp transaminase, the first step in the IAA biosynthetic pathway, and is the substrate for indolepyruvate decarboxylase. Indole-3-pyruvic acid is the AHR protagonist [83].

#### 3.10.3. IPA

TrpRS inhibitor indole-3-propionic acid (IPA, synonyms: 3-(3-Indolyl)propanoic acid, 3-Indolepropionic acid, β-(3-Indolyl)propionic acid; β-Indole-3-propionic acid) is a plant hormone with numerous cell growth functions. The plasma metabolomics revealed effects of antibiotic treatment on both IAA and IPA in rats [84]. Production of IPA was shown to be completely dependent on the presence of gut microflora and can be established by colonization with the bacterium *Clostridium sporogenes* [85]. Higher IPA is associated with reduced low-grade inflammation [86].

#### 3.10.4. d-Tryptophan

TrpRS potent inhibitor d-tryptophan was recently identified in probiotic bacteria [87]. d-tryptophan and d-phenylalanine exhibited toxic effects on *E. coli* growth [88]. Transport of d-tryptophan occurs via the amino acid transporter expressed predominantly in the distal intestine [89]. The diet contains both processing-induced and naturally formed d-amino acids [90,91].

#### 3.10.5. Indoleacrylic Acid (IAcrA)

In metabolomics study, IAcrA is increased two-fold while Trp decreased ~1.2-fold in plasma of AD versus cognitively normal (CN) [2]. A plant growth hormone IAcrA is an inhibitor of TrpRS [92,93] and tryptophan synthetase [94] and a marker of cardiotoxicity [95]. Commensal *Peptostreptococcus* species produce the Trp metabolite IAcrA [96]. IAcrA upregulates the gene expression of Trp operon in *E. coli* [97]. IAcrA has two effects: it prevents the Trp repressor from acting and it inhibits the charging of tRNA^Trp^ by TrpRS. The TrpRS inhibition by IAcrA [93], which is increased in AD plasma [2], may be sufficiently great to cause a significant decrease in the availability of charged tRNA^Trp^. The lowered concentrations of charged tRNA^Trp^ may restrict ribosome movement at tryptophan codons, thereby uncoupling transcription and translation.

#### 3.10.6. 3-Methylindole (3MI)

Trp decreased 1.229-fold and its product, the pulmonary toxin 3MI, increased 1.469-fold in the plasma of AD versus mild cognitive impairment (MCI) [2]. The 3MI (skatole) is produced from IAA by mixed populations of pig fecal bacteria [98] and also by *Clostridium drakei* and *Clostridium scatologenes* [99]. 3MI presents in the intestine of humans and pigs, causes acute pulmonary edema and emphysema [100] and induces the expression of AHR [101]. The olfactory impairment was correlated with olfactory neuronal populations in mice treated with 3MI [102]. The 27 compounds were screened for their ability to reduce the conversion of Trp to 3MI. Some of them reduced 3MI production by more than 80%. At least part of the inhibition occurs at the level of Trp conversion to IAA [103]. 3MI is a putative TrpRS inhibitor as the analog of its substrate.

### 3.11. Acetyltryptophan in Macular Degeneration

In patients with neovascular age-related macular degeneration (NVAMD), the increased levels of small peptides were detected in the plasma. This could be the result of excessive proteosomal activity, abnormalities in removal of peptides by peptidases or altered function of peptide transporters. Additional features in NVAMD are modified amino acids acetylphenylalanine and acetyltryptophan. The human plasma acetyltryptophan is higher and detected in elevated tripeptides in patients with NVAMD versus similarly-aged controls [104]. The acetyltryptophan is a TrpRS inhibitor [46] (Table 1). Therefore, we suggest that the increased level of acetyltryptophan-containing tripeptides could be the result of excessive degradation of abnormal proteins, containing modified Trp while elevated free acetyltryptophan may inhibit the TrpRS-dependent protein biosynthesis. *Aspergillus nidulans* reveals the non-ribosomal peptide biosynthetic pathway to acetylate Trp [105]. Acetyltryptophan and tryptophan were 2.4-fold and 1.7-fold, respectively higher in germ-free compared to conventional mice [85].

### 3.12. Di- and Tripeptides in AD, Mild Cognitive Impairment (MCI) and Cognitively Normal (CN)

In Table 2, we summarize the data on the levels of di- and tripeptides in plasma and cerebrospinal fluid (CSF) from the metabolomics study conducted by the Mayo clinic [2].

In plasma and CSF of patients with AD and MCI (Table 2), the levels of di- and tripeptides are altered compared to CN. We suggest that some of these short peptides can serve as markers: Glu-Ser is increased in AD and MCI plasma and decreased in AD and MCI CSF; Trp-Gly-Phe is decreased in AD/CN and AD/MCI plasma; Met-Trp-Gln (MWQ) is decreased in AD/CN and MCI/CN plasma; Pro-Lys-Pro is decreased in AD/CN and AD/MCI in CSF. We selected human proteins containing tripeptides altered in AD and/or MCI compared to CN by using NCBI database and amino acid sequences designed for tetra- or penta-peptides as a search tool such as KMWQ or SMWQY (Tables S1–S4). Using this search approach we revealed that human cytoskeleton protein axonemal dynein contains 11 tripeptides (Table S1) that are decreased in AD and/or MCI including two tripeptides MWQ. The Aβ oligomers are enriched in axons and interact with dynein motors. This interaction interferes with the coupling of the dynein motor with its adaptor SNAPIN [106], which is a Trp-free protein (Table 3).

**Figure 1:**
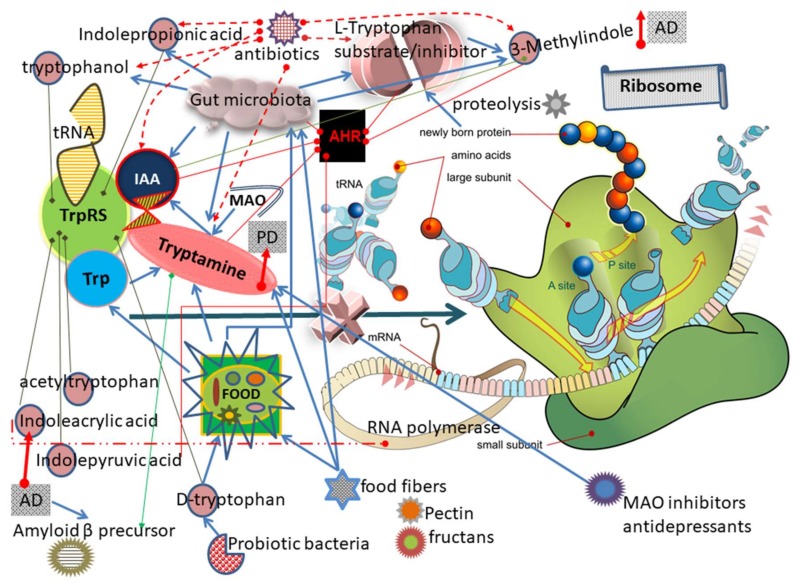
Scheme of inhibition and blockage of protein biosynthesis due to the replacement of Trp from TrpRS with Trp metabolites, the substrate-like inhibitors of TrpRS (abbreviations and explanations are in the text).

The increase in peptide levels could be the result of excessive proteosomal activity, abnormalities in removal of peptides by peptidases or altered function of peptide transporters. The decrease in peptide levels could be the result of tRNA aminoacylation blockage (Figure 1). The Figure 1 summarizes the data on TrpRS inhibition, tryptamine and Trp metabolites produced by gut microbiota as well as effects of the diet.

### 3.13. Link of Trp Frequency in Proteins to AD and Related Disorders

In the previous report, the author analyzed the frequency of Trp in proteins implicated in AD and related disorders [4]. Here we update and summarize the data on Trp-free proteins (Table 3). The analysis of Trp frequency and Trp position in proteins can shed light on a role of protein biosynthesis in protein abnormalities revealed in AD and related disorders. The average protein contains ~1.3% Trp (UGG nuclear codon). Some human proteins contain little or no Trp. Insufficient amount of aminoacylated tryptophanyl-tRNA^Trp^ for translating Trp-containing peptides can cause protein deficiency while Trp-free proteins might appear as overproduced in comparison with the reduced proteins containing Trp. Here is the example of such a scenario: one of 13 COX subunits, a human COX subunit VI-c of 7 kDa (75 aa) contains no tryptophan (Swiss-Prot: P09669.2). The protein level of this subunit is higher than a level of citrate synthase (9 Trp) in temporal cortex of AD; occipital cortex of Spinocerebellar Ataxia Type I; cerebellar cortex, frontal cortex and occipital cortex of Friedreich’s Ataxia than in control, whereas other COX subunits that contain Trp are decreased in neurodegenerative diseases [107]. The tryptophanyl-tRNA deficiency developed by using Trp metabolites [3] correlates with accumulation of Trp-free proteins in AD brain (Table 3). It was hypothesized [4] that under shortage of tryptophanyl-tRNA^Trp^, the proteins and polypeptides containing no Trp are relatively overrepresented in organs and/or fluids of patients in comparison with underrepresented deficient proteins containing Trp. In such a scenario, the Trp-free proteins become easily accessible for detection and isolation from organs and bodily fluids of patients. Some Trp-free proteins such as tau protein and synuclein are able to aggregate and form insoluble fibrils. The author focused mainly on the sequences of human proteins in the analysis of Trp content. The author suggests that the accumulation of tryptophan-free proteins such as tau protein is not a cause of disease but a consequence of TrpRS inhibition in disease.

### 3.14. Ribosomal Frameshifting and Bypassing

If there is deficient tryptophanyl-tRNA, the ribosome will be arrested at the Trp codon before it gets to the stop signal (Figure 1). However, there are adaptation mechanisms that help a paused ribosome in cells. Ribosomal frameshifting, particularly +1 frameshifts, can occur as a translational error. Certain codons, if they become ‘hungry’ through aminoacyl tRNA limitation, can frameshift in error to generate a nonsense product [108]. The potential role of ribosomal frameshifting in generating aberrant APP under neurodegeneration is suggested. Tryptamine treatment leading in tryptophanyl-tRNA^trp^ limitation can stimulate frameshifting of ribosomal translation and consequent generation of aberrant proteins. The Aβ_1–42_ polypeptide is a product of carboxy-terminal proteolysis (Asp672–Ala713) of full-length APP_770_ (Swiss-Prot: P05067). The alternatively spliced APP mini (278 aa) carboxyl form (GenBank: BAH12049), contains single Trp. The shifty APP_278_ mRNA sequence (5′CCC**GUGA**AUG**GAGAG**UUCAGCCUGGACGAUCUCCAGCCG**UGG**CAUUCUUUUGGG) includes the (−1) stop codon UGA in the (−1) frame and potential shifty site GA_GAG 24 nt 5′ of the Trp codon UGG. A single Trp of mini APP_278_ corresponds to Trp621 of the full-length APP_770_. Single Trp of APP_278_ can be removed by frameshifting to cause degradation of the unstable mutant protein into Trp-free Aβ (Table 3).

### 3.15. Trp Levels Decreased in the Human Bodily Fluids of Diseased Individuals

Total Trp levels were found to be diminished in plasma and CSF of multiple sclerosis, motor neuron disease and ataxia [109]. Protein-bound plasma Trp was lower in epileptic patients compared to normal volunteers [110]. In the metabolomics conducted by Mayo clinic, the levels of aminoacylated tRNA was altered in plasma and CSF of AD and MCI [2]. The enzymatic activity of TrpRS can be reduced as a result of inhibition by d and l tryptophan and its metabolites (Figure 1), or hypoxia-induced downregulation of the TrpRS gene expression [35], or TrpRS self-aggregation [36]. The reduction in tRNA^trp^ aminoacylation ultimately leads to the inhibition and blockage of protein biosynthesis (Table 2).

### 3.16. TrpRS Inhibition Leads to Both Translation and Transcription Impairments

In *Salmonella typhimurium*, the IAcrA usage to de-repress the trp operon results in a decline in RNA polymerase electron microscopic density observed on promoter-distal portions of cloned trp operons. This may be attributable to premature transcription termination accompanying translation inhibition due to the IAcrA interference with normal TrpRS activity [93]. In other words, electron microscopy directly demonstrates the decrease in RNA polymerase induced by TrpRS inhibitor. Therefore, TrpRS inhibition leads to both translation and transcription impairments. Using BLAST analysis we revealed that human DNA-directed RNA polymerase II can be decreased in AD since this enzyme contains tripeptide Pro-Lys-Pro (PKP) decreased in AD CSF; tripeptides Ile-Ser-Lys (ISK) and Ala-Thr-Pro (ATP) decreased in MCI plasma (Table S4). Our data indicate that the protein biosynthesis impairment induced by TrpRS inhibition causes neuronal death and abnormal protein conformation intracellularly and extracellularly in the brain of tryptamine-treated mice [3]. This model mimics closely AD and related neurodegenerative disorders.

### 3.17. Mutations in Genes Encoding Cytoplasmic (WARS) and Mitochondrial (WARS2) TrpRS in Humans

The experimental models of TrpRS deficiency are supported by recent discoveries of mutations in gene encoding mitochondrial TrpRS (WARS2) that implicate in intellectual disability, delayed myelination, seizures, whereas de novo synthesis of proteins inside mitochondria was reduced in the patient’s fibroblasts [111,112,113]. In a report by Musante et al., the p.Trp13Gly variant was found to significantly reduce the level of WARS2 protein in the mitochondrial fraction. Burke et al. reported biallelic mutations in WARS2 with reduced level of WARS2 protein that cause the levodopa-responsive infantile-onset parkinsonism [114]. In the distal hereditary motor neuropathy (DHMN), a heterozygous mutation was identified in the cytoplasmic TrpRS gene (WARS) that co-segregates with the neuropathy in the family. This mutation has a dominant negative effect on aminoacylation activity of TrpRS, which subsequently compromised protein synthesis and reduced cell viability [115]. DHMN characterized by degeneration and loss of motor neuron cells in the anterior horn of the spinal cord and subsequent muscle atrophy. Mutations in genes encoding other members of aminoacyl-tRNA synthetase family (prolyl-tRNA synthetase and asparaginyl-tRNA synthetase) are also related to infantile-onset neurodegenerative disorder [116].

### 3.18. Bacterial Toxic Analog of Amino Acid Alanine Causes Neurodegeneration in Monkey

The chronic dietary exposure of vervets (Chlorocebus sabaeus) to a cyanobacterial toxin present in the traditional Chamorro diet, beta-*N*-methylamino-l-alanine (BMAA), triggers the formation of both NFT and Aβ deposits similar in structure and density to those found in brain tissues of Chamorros who died with Guamanian amyotrophic lateral sclerosis/parkinsonism dementia complex [117]. Although BMAA is a putative inhibitor of aminoacyl-tRNA synthetase, the inhibitory activity for BMAA is unknown.

### 3.19. TrpRS, Trp and Tryptamine in Vascular Dysfunctions and Pathological Changes

**Figure 2:**
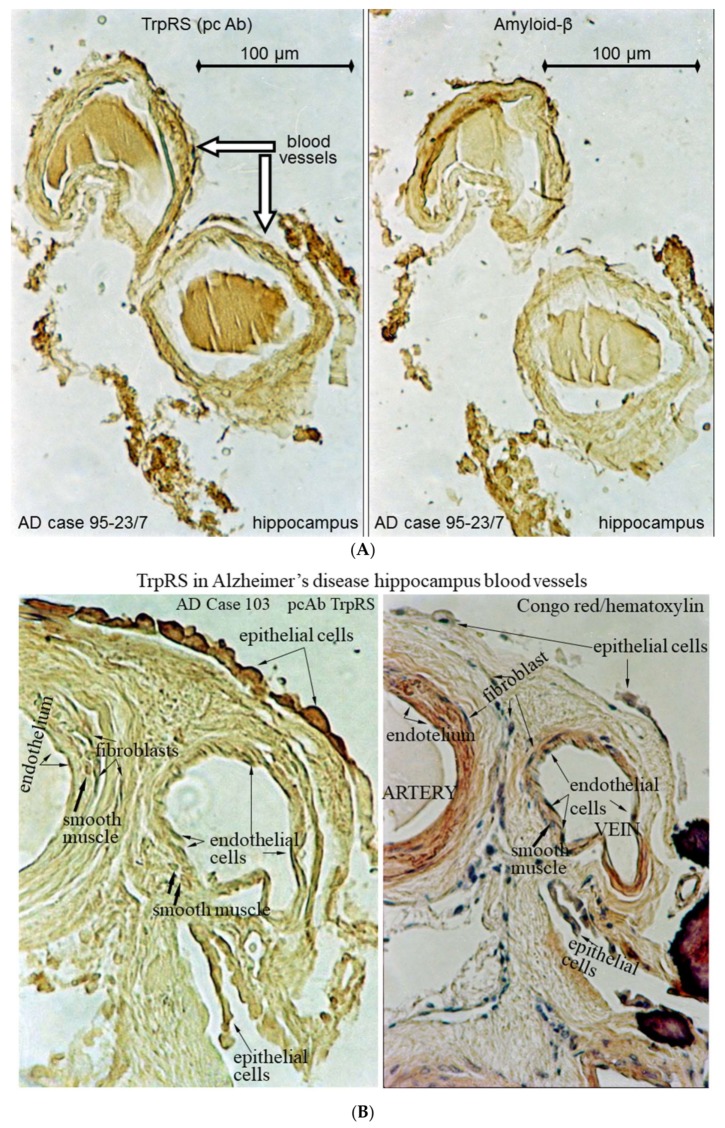

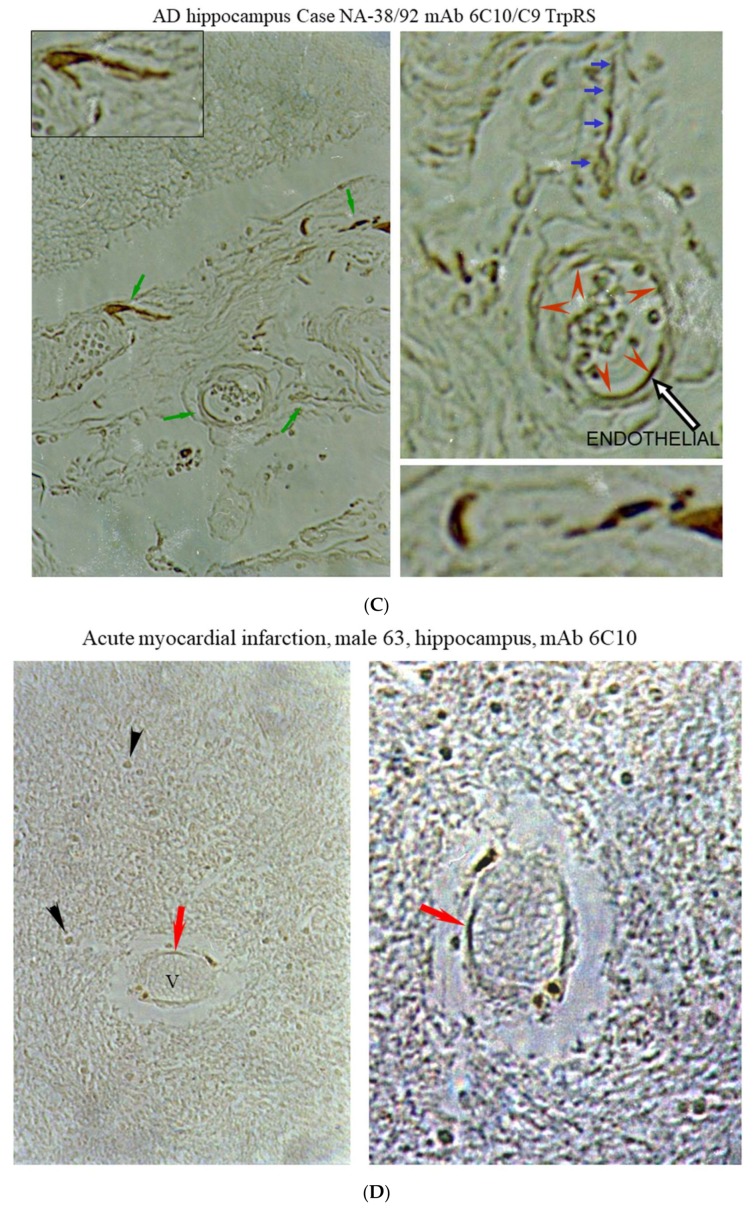

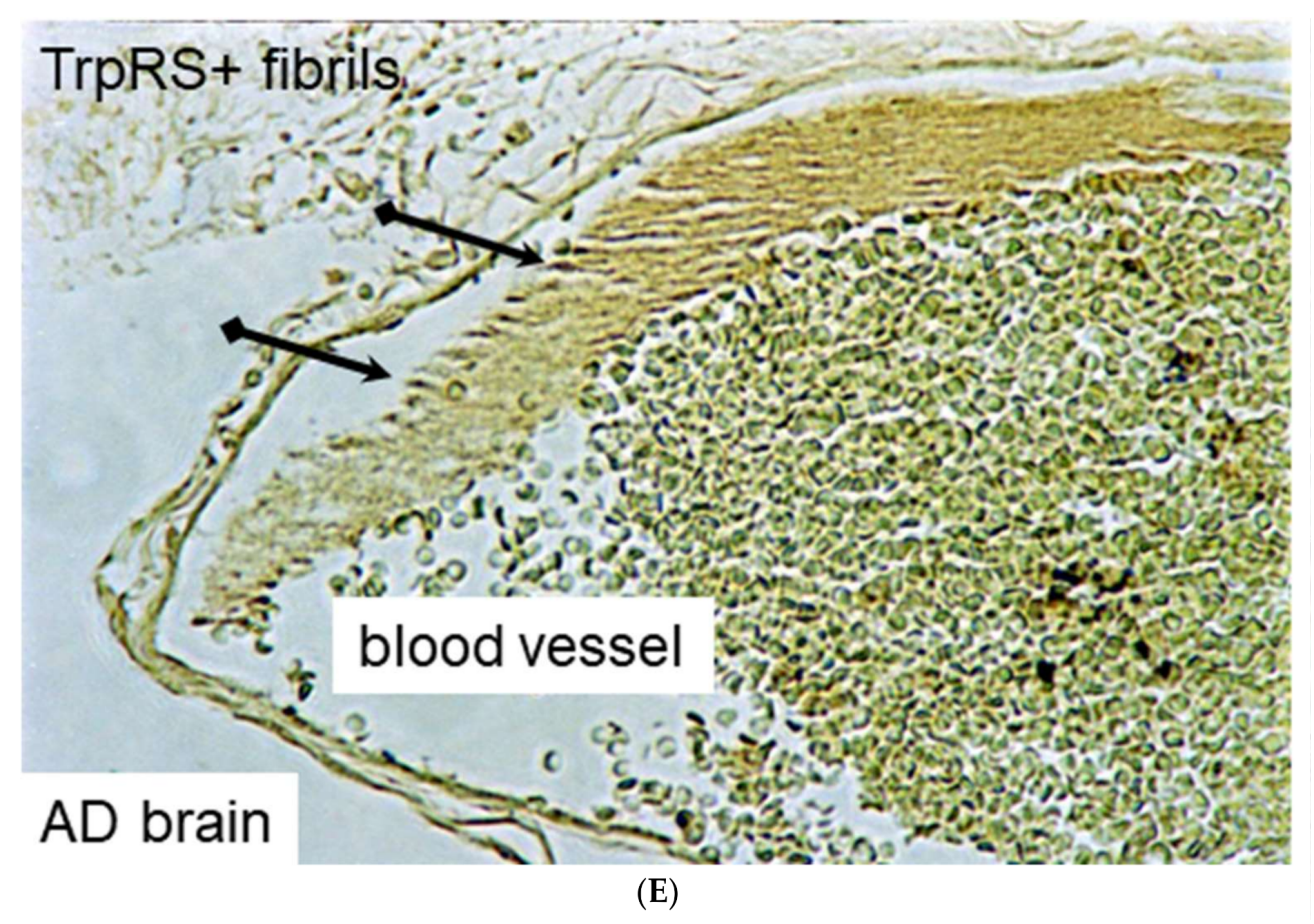
TrpRS is associated with AD vasculopathies. (**A**) The images demonstrate a partial co-localization of TrpRS (left panel) and Aβ (right panel) in the serial sections immunostained with anti-TrpRS antibodies (Ab) or Ab to Aβ. The clot-like TrpRS deposition is visible inside the vessel. The Aβ deposition is visualized in a neighboring section; (**B**) The blood vessels in the serial sections stained with Congo red/hematoxylin (right panel) or anti-TrpRS Ab (left panel); (**C**) Immunostaining of TrpRS in the blood vessel endothelial cells of AD hippocampus with mAb 6C10; (**D**) Immunostaining of TrpRS in the blood vessel endothelial cells of acute myocardial infarction hippocampus with mAb 6C10; (**E**) Microscopy of AD cerebral blood vessel immunostained with anti-TrpRS Ab. Arrows show the TrpRS immunostaining associated with fibrils inside the blood vessel.

**Figure 3:**
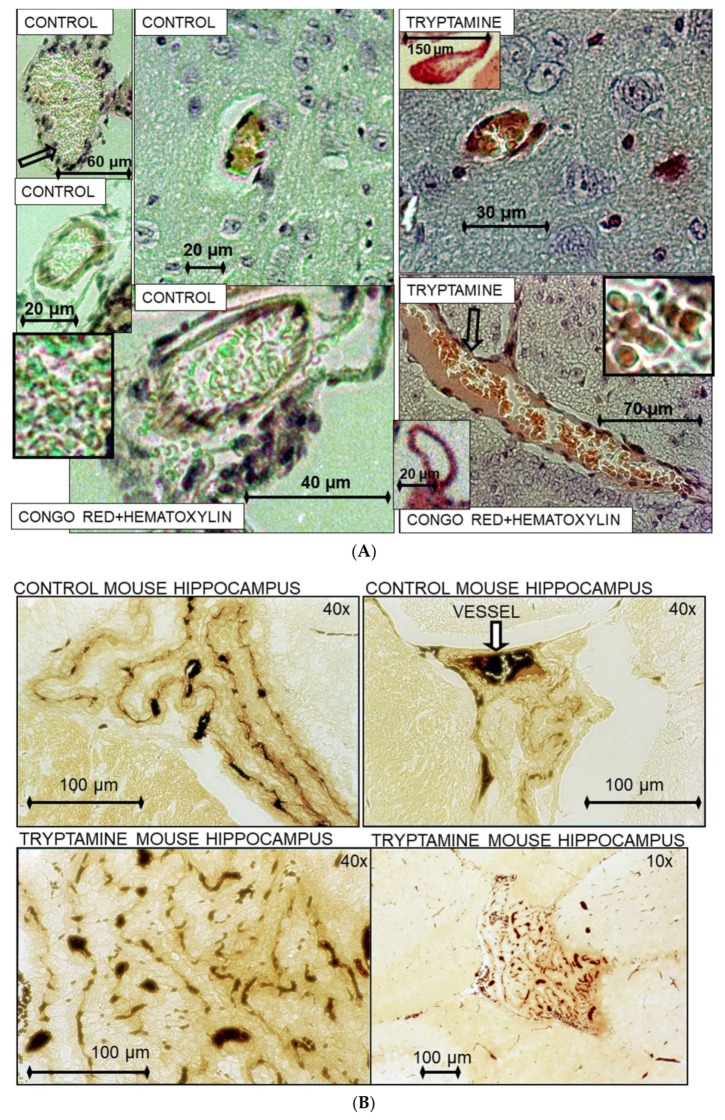
Vasculopathies induced by tryptamine in the hippocampal area of mouse brain. (**A**) The congophilic cerebral angiopathy in blood vessels of tryptamine-treated mouse; (**B**) The Gallyas silver staining shows the evidence of tryptamine-induced angiogenesis.

Cerebral amyloid angiopathy is common in AD and may contribute to dementia and cerebral hemorrhage. In human gut metagenome study, the aminoacyl-tRNA biosynthesis was altered in symptomatic atherosclerosis [118]. Vascular dementia (VaD) is the most common cause of dementia in the elderly, second to AD. The VaD cause or main risk factors are hypertension, diabetes mellitus, atherosclerosis, coronary artery disease [119]. In spinal cord injury in rats, application of physiological concentrations of Trp, tyrosine or phenylalanine (10–100 μM) induced tonic local constrictions of capillaries leading to a decrease in capillary diameter, which should severely impair blood flow. The Trp-induced constrictions produced changes in vessel morphology and widespread displacement of red blood cells. Tryptamine produced from Trp by aromatic amino acid decarboxylase induces similar vasoconstriction [120]. Tryptamine increases blood pressure by vasoconstriction and implicates in cardiovascular pathologies, including hypertension, migraine and myocardial infarction [121,122,123,124]. It has been shown that tryptamine (1–5 mg/kg, i.v.) decreases the resistance of the cerebral blood vessels and lowers the velocity of the blood flow and pO_2_ in the brain tissues of cats [125]. Here, we demonstrate the TrpRS depositions in the cerebral blood vessels of AD patients (Figure 2) and effects of tryptamine on cerebral blood vessels in mice (Figure 3).

In AD human brain, TrpRS was mainly visualized in association with congophilic angiopathy, as blood clot-like depositions inside the vessels and in the endothelial, epithelial and fibroblast cells (Figure 2A,B). The strong immunostaining of endothelial cells periphery with anti-TrpRS monoclonal antibody (mAb) can be an indicator of intensive TrpRS secretion (Figure 2C). The significant immunostaining of the endothelial cells with anti-TrpRS mAb was revealed in some blood vessels of hippocampus in acute myocardial infarction (Figure 2D). We did not reveal such vessel immunostaining in the brain serial sections immunostained with the secondary antibodies. The microscopy visualizes the fibrils immunostained with anti-TrpRS antibodies inside the AD cerebral blood vessel (Figure 2E). We show that tryptamine causes vasculopathies in mouse hippocampus including congophilic angiopathy (Figure 3A) and angiogenesis (Figure 3B). It was reported earlier that in the AD hippocampus, the ongoing angiogenesis results in increased vascular density compared with controls [126]. To our knowledge, this is the first study that demonstrates the vasculopathies induced by tryptamine in the hippocampus. Human trace amine receptors (including TAAR1, TAAR2, TAAR5, TAAR6, TAAR8, TAAR9) are expressed in the brain and play significant physiological and neuropathological roles by activation of trace amines including tryptamine [127].

## 4. Conclusions

Our analysis reveals that the tryptophan-containing tri-peptides diminished in the mild and severe AD compared to the normally cognitive individuals. This report demonstrates for the first time that the dietary product tryptamine induces vasculopathies in the hippocampus of mouse brain at the concentrations that inhibit TrpRS, while TrpRS is associated with vasculopathies in AD brain. The mutations in genes encoding cytoplasmic (WARS) and mitochondrial (WARS2) TrpRS enzymes cause the TrpRS deficiency, intellectual disability and Parkinsonism. Tryptamine producing microbes including bacteria and fungi present in most humans and in food. Tryptamine production in the human microbiome can be increased by a number of factors including antibiotics, dietary supplements and probiotics. Altogether, the data presented here indicate that the inhibition of TrpRS by tryptamine and other Trp metabolites cause the protein biosynthesis impairment with consequent neurodegeneration.

### Lethal Tryptamine Drug Combinations.

Most antidepressants—the MAO inhibitors raise tryptamine. When levels get too high, serious complications can occur including hypertension and seizures. In severe cases, this can even lead to death.

## Figures and Tables

**Table 1:** Reversible substrate-like inhibitors of bovine Tryptophanyl tRNA Synthetase (TrpRS) [46].

Inhibitor	*K_i_* (M)
5,7-Difluorotryptophan	2 × 10^−5^ ± 0.5 ^a^
4,5,6,7-Tetrafluorotryptophan	1.2 × 10^−5^ ± 0.3 ^a^
d-Tryptophan	5 × 10^−5 a^
Tryptamine	6 × 10^−5 a,#^
β-Indolylacetic acid	9 × 10^−3 b^
β-Indolylpropionic acid	8.5 × 10^−3 b^
β-Indolylpyruvic acid	5 × 10^−4 b^
*N*-Formyl-l-tryptophan	4.6 × 10^−4 b^
*N*-Acetyl-l-tryptophan	2.5 × 10^−4 b^
Adenine	1.8 × 10^−2 a^
Adenosine	3.1 × 10^−3 a^

^a^ In the reaction of ATP[^32^P]pyrophosphate exchange; ^b^ In the reaction of tRNA^trp^ charging; ^#^ Note, the Km value of purified bovine pancreatic TrpRS for tryptophan in ATP-[^32^P]pyrophosphate exchange (0.9 × 10^−7^ M) is six times lower than Ki for tryptamine (6.0 × 10^−7^ M) as reported [7,44]. In other studies, the tryptophan *K*_m_ for purified bovine pancreatic TrpRS is 1.4 ± 0.2 × 10^−7^ M [37] and 1.14 × 10^−6^ M for bovine kidney TrpRS in the cell extract [44] in the reaction of ATP-[^32^P]pyrophosphate exchange.

**Table 2:** The di- and tripeptides altered in plasma and CSF of AD and MCI.

AD/CN PLASMA	AD/MCI PLASMA	AD/CN CSF	AD/MCI CSF	MCI/CN PLASMA	MCI/CN CSF
Increase					
Met His Lys *	Gly His	Pro Pro ^$^	Tyr Pro ^§^	Ala Leu ^##^	Pro Pro ^$^
Val Ser Lys	Arg Asn Gln	Tyr Tyr Thr	Asn Gly Ser	Trp Ala Ile ^©^	Gln Pro Lys
Phe Ala Arg				Phe Val Val	
Met Glu Cys				Thr Ser Gln	
Thr Ser Gln				Glu Ser ^#^	
Glu Ser ^#^					
Val Gly					
Decrease					
Cys Tyr Cys	Trp Gly Phe ^©!^	Ala Phe Arg	Pro Lys Pro **	Ile Ser Lys	Leu Leu Ala
Trp Gly Phe ^©!^	Ala Leu ^##^	Pro Lys Pro **		Asp Asn Glu	Asn Gln Gln
Ser Asp Gly		Thr Gly		Leu Glu Gln	Ala Met Lys
Met Trp Gln ^©!^		Asp Glu		Met Trp Gln ^©!^	Glu Ser ^#^
Met His Lys *		Glu Ser ^#^		Ala Thr Pro	Ala Ala Asp
		Cys Cys Tyr			Tyr Pro ^§^
		Arg Cys Cys			
		Met Ala His			

AD—Alzheimer’s disease; MCI—mild cognitive impairment; CN—cognitively normal; CSF—cerebrospinal fluid (the data compiled based on Supplemental Tables S1–S10 of Trushina et al., PLOS, 2013, Mayo clinic study, metabolomics data [2]. Trp is a rarest (~1.3%) amino acid, Ser is the most frequent (8.1%) amino acid in proteins. Black letters—increase, white letters—decrease, ^©^ Trp-peptides; ^!^ decrease in Trp possessing tripeptides; *, ^#^, ^$^, ^§^, ^##^, ** repeat.

**Table 3:** Human Trp-free proteins and polypeptides.

Protein/Peptide	Amino Acids	Function	Database ID
COX subunit VI-c	75	electron transfer	Swiss-Prot: P09669.2
tau protein	758	microtubule-associated	Swiss-Prot: P10636
islet amyloid peptide	89	pro-amylin glycemic	GenBank: AAA52281
beta-amyloid peptide	40	not understood	Swiss-Prot: P86906.1
prion protein	108	controversial	PDB: 1I4M_A
alpha-synuclein	140	not understood	GenBank: NP_000336
beta-synuclein	134	unknown	GenBank:NP_001001502
gamma-synuclein	127	unknown	GenBank: AAL05870
collagen, type XXV	645	AD plaque component	GenBank:EAX06240
ubiquitin	156	regulation	GenBank: CAA44911
S100B	92	regulation	GenBank: CAG46920
histone H2A	130	chromatin structure	GenBank: CAA58539
histone H3	136	chromatin structure	GenBank: CAB02546
neurofilament medium	540	cytoskeleton	GenBank:NP_001099011
myelin basic protein	160	myelination	GenBank: NP_001020263
arrestin	409	signal trunsduction	GenBank: CAA77577
TATA box binding	338	transcription factor	GenBank: AAI09054
calcitonin	141	hormone	GenBank: NP_001029124
thyroid hormone	138	stimulating hormone	GenBank: AAH69298
glycoprotein hormones	116	hormone	GenBank: NP_000726
oxytocin	125	hormone	GenBank: AAI01844
arginine vasopressin	164	hormone	GenBank: AAI26197
prothymosin alpha	111	immunity	GenBank NP_001092755
snapin	136	synaptic transmission	GenBank: AAD11417.1
Interleukin-9	144	cytokine	GenBank: AAH66284.1
interleukin-18	189	Increased in AD	NCBI: NP_001230140.1
epidermal growth factor	71	Increased in AD	GenBank: CAA34902.2
interleukin-2 isoform X1	131	lymphokine	NCBI: XP_016863666.1
C-X-C motif chemokine 10 precursor	98	Cytokine, elevated in AD	NCBI: NP_001556.2
NADH dehydrogenase (ubiquinone) flavoprotein 3	108	mitochondrial isoform b Renal carcinoma antigen NY-REN-4	NCBI: NP_001001503.1
NADH dehydrogenase (ubiquinone) flavoprotein 3	473	mitochondrial isoform a precursor	NCBI: NP_066553.3
NADH dehydrogenase (ubiquinone) iron-sulfur	124	mitochondrial precursor protein 6	NCBI: NP_004544.1
NADH dehydrogenase	119	NADHDH2	GenBank: AAP97198.1
NADH dehydrogenase	210	Human gut metagenom	GenBank: EKC78685.1
NADH dehydrogenase	167	human gut metagenom	GenBank: EKC44884.1
syntaxin	259	synaptic vesicles REN31	PIR: G01485
syntaxin-2 isoform 3	277	synaptic vesicles	NCBI: NP_001337978
syntaxin-3 isoform 1	289	synaptic vesicles	NP_004168.1
GTPase HRas	189	regulating cell division	Swiss-Prot: P01112.1

## Supplementary Materials

The following are available online at http://www.mdpi.com/2072-6643/10/4/410/s1, Table S1: Proteins containing Met-Trp-Gln (MWQ) decreased in AD/CN and MCI/CN plasma, Table S2: Proteins containing Trp-Gly-Phe (WGF) decreased in AD/CN and AD/MCI plasma, Table S3: Proteins containing Pro-Lys-Pro (PKP) decreased in AD/CN and AD/MCI in CSF, Table S4: Human RNA polymerase subunits contain tripeptides decreased in AD or MCI.

## Appendix A

Dissociation constant (Kdis) for tryptamine in the tryptophan-dependent ATP-[32P] pyrophosphate exchange catalyzed by bovine pancreatic TrpRS after covalent binding of 1 mole of R-Trp-tRNA to the enzyme (one site of modified TrpRS was occupied after the binding) is 13 ± 4 × 10^−7^ M versus tryptamine Kdis 3.6 ± 0.6 × 10^−7^ M for the native enzyme [128]. The different affinities of the two subunits of bovine pancreatic TrpRS for tryptophan were observed by Graves et al. [129] and Mazat et al. [130]. Taking an initial symmetrical dimeric TrpRS the two dissociation constants for tryptophan at pH 8 and 25 °C are respectively K_1_ = 2.0 ± 0.5 µM and K_2_ = 10 ± 4 µM [129]. They are respectively K_1_ = 1 ± 0.25 µM and K_2_ = 20 ± 8 µM if one considers a sequenced binding of the two tryptophan molecules. The tryptamine Ki for L-tryptophan and the fluorinated substrate L-6-fluoro-tryptophan [131] were determined in ATP-[32P] pyrophosphate exchange catalyzed by bovine pancreatic TrpRS to be 3.2 ± 0.6 × 10^−7^ M and 1.7 ± 0.4 × 10^−7^ M, respectively [132].
