# Supplementary material for: Towards an Integrative Understanding of tRNA Aminoacylation–Diet–Host–Gut Microbiome Interactions in Neurodegeneration
Source: Nutrients. 2018 Mar 26;10(4):410. doi: 10.3390/nu10040410 (PMC5946195; doi:10.3390/nu10040410)
Supplement: Supplementary file 1 [file nutrients-10-00410-s001.pdf]

nutrients

**Supplementary Materials:**

**Table S1.** Proteins containing Met-Trp-Gln (MWQ) decreased in AD/CN and MCI/CN plasma

| Human Protein | Sequence ID |
|---|---|
| glutaminyl-peptide cyclotransferase precursor | NP_036545 |
| anchor protein | AAU94938.1 |
| cadherin EGF LAG seven-pass G-type receptor 3 precursor | NP_001398.2 |
| telomerase protein component 1 isoform 1 | NP_009041.2 |
| dedicator of cytokinesis protein 3 | NP_004938.1 |
| TSNARE1 protein | AAI43687.1 |
| marginal zone B- and B1-cell-specific protein precursor | NP_057543.2 |
| solute carrier family 22 (organic cation transporter). N-terminal Met-Trp-Gln | EAX02529.1 |
| gem (nuclear organelle) associated protein 5, isoform CRA_b | EAW61624 |
| DOCK4 (GTPase activator) | AAO73565.1 |
| catenin delta-2 isoform 1 | NP_001323 |
| integrin alpha subunit | BAA04984.1 |
| acetyltransferase | AAB62398.1 |
| immunoglobulin light chain variable region | AIE57082.1 (*KMWQ) |
| immunoglobulin kappa chain | AAA99330.1 (KMWQ) |
| phosphodiesterase 1A, calmodulin-dependent | EAX10972.1 (KMWQ) |
| calcium channel, voltage-dependent | EAW84361.1 |
| neuronal calcium channel alpha 1A subunit | AAB61613.1 |
| regulator of G-protein signaling like 2 | EAW91126.1 (KMWQ) |
| alpha-1-antitrypsin-related protein | AGI62067.1 (KMWQ) |
| chromosome 9 open reading frame 79 | EAW62739.1 (KMWQ) |
| spermatogenesis-associated protein 31E1 | NP_849150.3 (KMWQ) |
| phospholipase C epsilon | AAG28341.1 (KMWQ) |
| nuclear ribonucleoprotein 200 kDa helicase | NP_054733.2 (RMWQ) |
| claudin-5 | NP_003268.2 (PMWQ) |
| ADP-ribosyltransferase 4 | CEK43034.1 (RMWQ) |
| putative NFkB activating protein | BAC77374 (PMWQ) |
| SCO-spondin precursor | NP_940857.2 (AMWQ) |
| dynein heavy chain 3, axonemal isoform 1 | NP_060009.1 (SMWQ) (VMWQ) |
| delta-catenin | GenBank: AAC63103.1 (SMWQY) |
| ras-related protein Rab-18 | NP_001243339.1 |
| TATA binding protein associated factor | AAC68502.1 |

*The tetra- or pentapeptides (examples in parentheses) containing altered Trp-containing tripeptides were used in the BLAST search as described in Materials and Methods. Amino acids in peptides are shown with one letter code

**Table S2.** Proteins containing Trp-Gly-Phe (WGF) decreased in AD/CN and AD/MCI plasma.

| Human Protein | Database ID |
|---|---|
| mediator of RNA polymerase II transcription | GenBank: EAW78801.1 |
| urea transporter | NCBI: NP_001139509.1 |
| cholesteryl ester transfer protein | GenBank: AAB59388.1 |
| multiple epidermal growth factor-like domains | NCBI: NP_001258867.1 |
| ADAM metallopeptidase domain | GenBank: EAW51578.1 |
| JAK family protein tyrosine kinase | GenBank: AAA19626.1 (KWGF) |
| IFN-tyk, tyk2=interferon alpha/beta signaling | GenBank: AAB22747.1 (KWGF) |
| secretory phospholipase A2 receptor | NCBI: NP_001007268.1 (KWGF) |
| Synaptopodin 2 | GenBank: AAI50630.1 (PWGF) |

**Table S3.** Proteins containing Pro-Lys-Pro (PKP) decreased in AD/CN and AD/MCI in CSF.

| Human Protein | Sequence ID |
|---|---|
| histone-lysine N-methyltransferase 2A | NP_005924.2 (KPKP*) 3 × PKP ** |
| protocadherin Fat 1 precursor | NP_005236.2 (KPKP) |
| low-density lipoprotein receptor-related | XP_011509485.1 (KPKP) |
| ubiquitin-conjugating BIR-domain enzyme | AAF75772.1 (KPKP) |
| titin | ACN81321.1 (KPKP) 12 × PKP |
| B double prime 1, subunit of RNA polymerase III transcription initiation factor IIIB | AAI46793.1 (KPKP) 4 × PKP |
| zinc finger protein 40 isoform X3 | XP_011512857 (KPKP) |
| adlican | AAF86402.1 (KPKP) |
| kalirin Huntingtin-associated -interacting protein | XP_016862918.1 (PKP) |
| transcriptional activator SRCAP | AAD39760.1 (RPKP) |
| histone-lysine N-methyltransferase 2D isoform X6 | XP_006719677.1 (5 × PKP) |
| obscurin isoform X1 | XP_016857932.1 |
| DNA-directed RNA polymerase II | P24928.2 |
| dynactin | GenBank: CAA67333.1 (RPKP) |

* The peptides in parentheses were used in BLAST search as described in Materials and Methods.
** Some proteins contain more than one tripeptide altered in neurodegeneration; for instance titin contains 12 PKP peptides (12 × PKP).

**Table S4.** Human RNA polymerase subunits contain tripeptides decreased in AD or MCI.

| RNA polymerase | Sequence ID | Tripeptides Decreased in AD, MCI |
|---|---|---|
| RNA polymerase II subunit RPB1 | P24928.2 | PKP * (AD/CN, AD/MCI), ISK * and ATP * (MCI/CN) |
| RNA polymerase III subunit RPC1 | NP_008986.2 | AAD (MCI/CN), MAH (AD/CN) |
| RNA polymerase III subunit RPC2 | NP_060552.4 | SDG (AD/CN), LLA × 3 (MCI/CN) |

* Amino acids in tripeptides are shown with one letter code.

© 2018 by the authors. Submitted for possible open access publication under the terms and conditions of the Creative Commons Attribution (CC BY) license (http://creativecommons.org/licenses/by/4.0/).
